# Hydrophilic bile acids protect human blood-brain barrier endothelial cells from disruption by unconjugated bilirubin: an *in vitro* study

**DOI:** 10.3389/fnins.2015.00080

**Published:** 2015-03-13

**Authors:** Inês Palmela, Leonor Correia, Rui F. M. Silva, Hiroyuki Sasaki, Kwang S. Kim, Dora Brites, Maria A. Brito

**Affiliations:** ^1^Research Institute for Medicines (iMed.ULisboa), Faculdade de Farmácia, Universidade de LisboaLisbon, Portugal; ^2^Department of Biochemistry and Human Biology, Faculdade de Farmácia, Universidade de LisboaLisbon, Portugal; ^3^Division of Fine Morphology, Core Research Facilities, The Jikei University School of MedicineTokyo Japan; ^4^Division of Infectious Diseases, Johns Hopkins University School of MedicineBaltimore, MA, USA

**Keywords:** blood-brain barrier, glycoursodeoxycholic acid, human brain microvascular endothelial cells, interleukin-6, unconjugated bilirubin, ursodeoxycholic acid

## Abstract

Ursodeoxycholic acid and its main conjugate glycoursodeoxycholic acid are bile acids with neuroprotective properties. Our previous studies demonstrated their anti-apoptotic, anti-inflammatory, and antioxidant properties in neural cells exposed to elevated levels of unconjugated bilirubin (UCB) as in severe jaundice. In a simplified model of the blood-brain barrier, formed by confluent monolayers of a cell line of human brain microvascular endothelial cells, UCB has shown to induce caspase-3 activation and cell death, as well as interleukin-6 release and a loss of blood-brain barrier integrity. Here, we tested the preventive and restorative effects of these bile acids regarding the disruption of blood-brain barrier properties by UCB in *in vitro* conditions mimicking severe neonatal hyperbilirubinemia and using the same experimental blood-brain barrier model. Both bile acids reduced the apoptotic cell death induced by UCB, but only glycoursodeoxycholic acid significantly counteracted caspase-3 activation. Bile acids also prevented the upregulation of interleukin-6 mRNA, whereas only ursodeoxycholic acid abrogated cytokine release. Regarding barrier integrity, only ursodeoxycholic acid abrogated UCB-induced barrier permeability. Better protective effects were obtained by bile acid pre-treatment, but a strong efficacy was still observed by their addition after UCB treatment. Finally, both bile acids showed ability to cross confluent monolayers of human brain microvascular endothelial cells in a time-dependent manner. Collectively, data disclose a therapeutic time-window for preventive and restorative effects of ursodeoxycholic acid and glycoursodeoxycholic acid against UCB-induced blood-brain barrier disruption and damage to human brain microvascular endothelial cells.

## Introduction

In neonatal life, increased levels and prolonged exposure to unconjugated bilirubin (UCB) may trigger bilirubin-induced neurological dysfunction (Cohen et al., 2010). Although the mechanisms underlying neurological dysfunction are still unclear, the understanding of UCB-induced neurotoxicity has increased greatly in the past years (Brites and Brito, 2012). A general impairment of membrane structure, properties, and function (Rodrigues et al., 2002b; Brito et al., 2004), with neuronal oxidative stress, the release of pro-inflammatory cytokines by glial cells and altered myelinogenesis have been demonstrated (Silva et al., 2002, 2010; Falcão et al., 2006; Fernandes et al., 2006; Brito et al., 2008; Vaz et al., 2010; Barateiro et al., 2014). The awareness of the important role of the blood-brain barrier (BBB) and particularly of brain microvascular endothelial cells (BMEC) in the course of bilirubin-induced neurological dysfunction has also grown. In fact, the influence of UCB on porcine and rat BMEC (Akin et al., 2002; Cardoso et al., 2012) and in a mouse BMEC line (Kapitulnik et al., 2012) revealed that UCB induces a loss of endothelial cell viability. Our recent studies on human BMEC (HBMEC) have shown that UCB decreases endothelial cell survival and induces the release of cytokines, such as interleukin-6 (Palmela et al., 2011), which are known to be involved in BBB disruption in pathological conditions (Kaur and Ling, 2008; Carvey et al., 2009). Furthermore, HBMEC exposure to UCB resulted in biphasic effects depending on the time of interaction, where prolonged incubation compromised the endothelial junctions and led to significant impairment of barrier integrity (Palmela et al., 2012). Interestingly, UCB-induced disruption of barrier properties of BMEC was observed even in the presence of astrocytes (Cardoso et al., 2012), an *in vitro* co-culture model that better resembles the *in vivo* condition. Importantly, these *in vitro* evidences have been confirmed in autopsy studies of a kernicterus premature infant presenting increased vascularization and infiltration of erythrocytes and albumin in the brain parenchyma (Brito et al., 2013). Moreover, recent studies of additional cases of kernicterus have shown that the most susceptible brain regions to UCB toxicity, as the cerebellum, hippocampus, and basal ganglia, present marked signs of BBB dysfunction, as reduced pericyte vascular coverage and alterations in the basement membrane (Palmela et al., submitted). Thus, these features point to an enhanced permeability of the vascular walls, at least in severely ill pre-term infants with bilirubin encephalopathy.

The bile acid ursodeoxycholic acid (UDCA), which exists in very low levels in the circulation in humans, is largely used as therapy for chronic liver diseases involving cholestasis (Poupon et al., 1994; Brites et al., 1998; Lazaridis et al., 2001). UDCA is conjugated in the liver originating tauroursodeoxycholic acid (TUDCA) and glycoursodeoxycholic acid (GUDCA), the latest accounting for approximately 80% of the bile acid conjugates produced in patients under therapy (Rudolph et al., 2002). In addition, a four-fold increase of GUDCA relatively to that of TUDCA was found in the bile of patients with complete extrahepatic biliary obstruction treated with UDCA (Rudolph et al., 2002). Several studies have suggested a potential role of UDCA in the treatment of non-liver diseases involving increased levels of apoptosis (Keene et al., 2002; Rodrigues et al., 2003) due to the anti-apoptotic properties of this bile acid (Amaral et al., 2009b). Interestingly, the anti-apoptotic properties of UDCA were recently demonstrated in osteoblasts exposed to bilirubin (Ruiz-Gaspa et al., 2014). Our own studies have shown that UDCA and GUDCA protect astrocytes from apoptosis and suppress the production of pro-inflammatory cytokines (Rodrigues et al., 2000; Silva et al., 2001b; Fernandes et al., 2007a) while also counteracting UCB-induced neuronal death and synaptic changes (Silva et al., 2012). Moreover, GUDCA abrogated UCB-induced alterations in the redox status, mitochondrial dysfunction and energy impairment in neurons (Brito et al., 2008; Vaz et al., 2010). Interestingly, the mechanism of action of UDCA and its conjugates appears to rely on the stabilization of the cell membrane structure and maintenance of its dynamic properties, derived from their ability to prevent alterations in membrane lipid polarity and fluidity, as well as in the protein order and redox status (Rodrigues et al., 2001, 2002b; Solá et al., 2002).

In regard to the beneficial effects of these bile acids on endothelial cells, little is known. Nevertheless, it was shown that TUDCA is able to protect against amyloid-β-induced apoptosis (Viana et al., 2009) and leukocyte rolling and adhesion to the endothelium induced by lipid peroxidation products (Vladykovskaya et al., 2012), as well as to promote vessel repair (Cho et al., 2015). Interestingly, UDCA was shown to inhibit endothelin-1 production (Ma et al., 2004) and to have an anti-angiogenic capacity (Suh et al., 1997; Woo et al., 2010), suggesting an influence on endothelial cells in a much more complex manner. However, it remains unknown whether protective effects of UDCA and GUDCA are exerted on the endothelial cells of the human BBB, and specifically toward UCB-induced injury. Thus, we here aimed to first evaluate if such bile acids are able to protect HBMEC from UCB-induced apoptosis and ultrastructural changes. Next, we intended to investigate if UDCA and GUDCA are able to prevent the production of a mediator of endothelial permeability, interleukin-6, as well as changes in barrier integrity induced by UCB. By treating cells with UDCA and GUDCA prior to UCB exposure, or 4 and 8 h after the initiation of the incubation procedure, our purpose was to establish the therapeutic window of opportunity to be used in jaundiced infants at risk of bilirubin-induced neurological dysfunction requiring complementary medicines to the conventional treatments.

## Materials and methods

### Chemicals

The basal medium Roswell Park Memorial Institute 1640, antibiotic-antimycotic solution, human serum albumin (fraction V, fatty acid free), bovine serum albumin, Hoechst 33258 dye, sodium fluorescein and UCB were purchased from Sigma Chemical Co. (St. louis, MO, USA). Non-essential amino acids, sodium pyruvate, l-glutamine, fetal bovine serum and minimum essential medium vitamins were from Biochrom AG (Berlin, Germany). Nuserum IV and rat-tail collagen I were acquired from BD Biosciences (Erembodegem, Belgium). TRIzol Plus RNA Purification Kit, was from Invitrogen (Carlsbad, CA, USA). Caspase-3 substrate and Ac-Asp-Glu-Val-Asp-*p*-nitroanilide, were acquired from Calbiochem (San Diego, CA, USA). DuoSet ELISA kit was from R&D systems (Minneapolis, MN, USA). Primers for real-time PCR analysis were purchased from Thermo Scientific (Soeborg, Denmark). RevertAid H Minus First Strand cDNA synthesis and Maxima SYBR Green qPCR Master Mix (2×) were obtained from Fermentas (Burlington, ON, Canada). All other chemicals were of analytical grade and were purchased from Merck (Darmstadt, Germany).

### Cell culture and treatment

To test whether UCB-induced injury to endothelial cells could be abrogated in the presence of UDCA and GUDCA, we used a HBMEC line as a simplified model of the human BBB. This cell line was derived from primary cultures of HBMEC transfected with SV40 large T antigen (Stins et al., 2001) and was recently proved to be the most suitable human cell line for an *in vitro* BBB concerning barrier tightness (Eigenmann et al., 2013). Cells were cultured in Roswell Park Memorial Institute medium supplemented with 10% fetal bovine serum, 10% NuSerum IV, 1% non-essential amino acids, 1% minimum essential medium vitamins, 1 mM sodium pyruvate, 2 mM l-glutamine, and 1% antibiotic-antimycotic solution, seeded at a density of 8 × 10^4^ cell/mL in collagen I-coated coverslips or plates and treated after 2 days in culture, as previously described (Palmela et al., 2011). For integrity studies, based on the measurement of paracellular permeability to sodium fluorescein, cells were seeded on collagen I-coated polyester transwell inserts (0.4 μm, Corning Costar Corp., USA) at a density of 8 × 10^4^ cell/insert and treated after 8 days in culture (Palmela et al., 2012). Endothelial cultures were maintained at 37°C in a humid atmosphere enriched with 5% CO_2_, and all experiments were performed at confluence.

UCB was purified (Mcdonagh and Assisi, 1972) and a 10 mM stock solution was prepared in 0.1 M NaOH and used immediately after preparation. The pH value was restored to 7.4 by addition of equal amounts of 0.1 M HCl, and all the procedures were performed under light protection to avoid photodegradation. Confluent monolayers of the HBMEC line were incubated with 100 μM UCB, or with no addition (control), in the presence of 100 μM human serum albumin. This experimental condition mimics the bilirubin/albumin ratio (1:1 molar ratio; 8.7 mg/g) found in a kernicterus case described by us (Brito et al., 2012) and is within the 5.4–21.0 mg/g ratio recently associated to acute bilirubin encephalopathy in Egypt (Iskander et al., 2014). Determination of unbound bilirubin, or free bilirubin, by the widely used peroxidase method (Roca et al., 2006) showed that this experimental condition corresponds to a free bilirubin concentration of 23.6 nM, as previously reported by Palmela et al. (2012). The free bilirubin level used in the present *in vitro* model is within the range of values found by us in a group of moderately jaundiced neonates (19.1 ± 1.5 nM) (Brito, 2001) and by Ahlfors et al. (2009) (21–51 nM) in babies readmitted for jaundice. Also to mention that the apparent discrepancy between the free bilirubin value obtained in our lab and those indicated by Roca et al. (2006) may result from the fact that Roca et al. (2006) did not include cells in their system, thus not considering the fraction of bilirubin that is bound/included in cells (Brito et al., 2000; Palmela et al., 2012), nor the non-conjugating pathways for UCB catabolism (Ahlfors et al., 2009). The incubation period used for each parameter varied between 1 and 48 h, based on the time to obtain the maximal effect observed in prior studies (Palmela et al., 2011, 2012). The incubation medium consisted in the regular medium without fetal bovine serum and Nuserum IV, to avoid disturbance of the final concentration of albumin in the incubation medium.

Co-incubation studies were also performed with the bile acids UDCA and GUDCA, molecules with octanol/water partition coefficients of 1000 for the unconjugated form and 105 for the glycine-amidated molecule, and logP values of 3.0 and 2.02 for the former and the later, respectively (Roda et al., 1990). In the co-incubation studies, UDCA or GUDCA were added at a final concentration of 50 μ M, which is found in the circulation of patients under therapy with UDCA. In particular, the concentration of 50 μM GUDCA is commonly found in the serum of patients after treatment with UDCA at a dose of 13–15 mg per kilogram of body weight per day (Podda et al., 1990; Poupon et al., 1994; Brites et al., 1998). We previously showed that such concentration is not toxic to neurons (Silva et al., 2001b) and, most importantly, has beneficial properties in preventing neurodegeneration (Brito et al., 2008; Vaz et al., 2010). The bile acids were added at three different time points: 1 h prior to UCB addition and at 4 or 8 h after UCB incubation. For short periods of UCB incubation only the effects of 1 h pre-incubation with the bile acids were evaluated. Appropriate controls including cells treated with UDCA and GUDCA (without UCB) were also included to ascertain the absence of toxicity of these molecules.

For the integrity experiments, endothelial cells were cultured on semipermeable filters inserted in the culture plate well. With this system, there are two compartments: the apical or upper one that can be considered as the “blood-side,” where UCB, bile acids and human serum albumin were added, and the basal or lower compartment, which is considered the “brain side.”

### Assessment of apoptosis

Caspase-3 activity and the number of apoptotic nuclei were determined after 4 and 48 h of UCB exposure, respectively, since these time points represent the maximum effects of UCB alone (Palmela et al., 2011).

Activity of caspase-3 was measured by a colorimetric method (Calbiochem, Darmstadt, Germany), as usual in our lab (Palmela et al., 2011). The results were expressed as fold change from control values. Assessment of nuclear morphology of the HBMEC line following Hoechst 33258 staining was evaluated as previously described (Palmela et al., 2011). Fluorescence was visualized using a Leica DFC 490 camera (Leica, Wetzlar, Germany) adapted to an AxioScope.A1 microscope (Zeiss, Göttingen, Germany). Values were expressed as percentage of apoptotic nuclei.

### Transmission electron microscopy

Ultrastructural analysis was performed by transmission electron microscopy following 48 h exposure to UCB in HBMEC pre-treated with UDCA or GUDCA. Cells were fixed with 1.2% glutaraldehyde in 0.1 M phosphate buffer and 1% osmium tetroxide in the same buffer, dehydrated with a graded series of ethanol, and then embedded in epoxy resin. Ultrathin sections were stained with uranyl acetate and lead citrate and observed with a Hitachi H-7500 transmission electron microscope (Tokyo, Japan) at an acceleration voltage of 80 kV.

### Measurement of interleukin-6 mRNA expression and protein release

The HBMEC line was exposed to UCB for 1 h for mRNA analysis, and for 4 h for cytokine release quantification, since the maximum effects of UCB alone in interleukin-6 expression and secretion were observed at these time points (Palmela et al., 2011).

Analysis of mRNA expression was performed by quantitative real time PCR using a SYBR Green qPCR Master Mix (2×), as described previously (Palmela et al., 2011). This assay was performed using β-actin as an endogenous control to normalize the expression level of interleukin-6 mRNA. The following sequences were used as primers: interleukin-6 sense, 5′-GACAGCCACTCACCTCTTCA-3′ and anti-sense, 5′-TTCACCAGGCAAGTCTCCTC-3′ (Wang et al., 2006); β-actin sense, 5′-ACAGAGCCTCGCCTTTGCCG-3′ and anti-sense, 5′-TGGGCCTCGTCGCCCACATA-3′ (NM_001101.3). Non-specific products of PCR were not found in any case. The relative quantification was made using the Pfaffl modification of the ΔΔC_T_ equation (C_T_, cycle number at which the fluorescence passes the threshold level of detection), taking into account the efficiencies of individual genes. The results were normalized to β-actin and the initial amount of the template of each sample was determined as fold change from control samples (reference).

Endothelial interleukin-6 release was assessed in duplicate, using a specific DuoSet ELISA development kit, according to the manufacturer's instructions. Measurements were obtained at a wavelength of 450 nm, with a reference filter of 620 nm, using a microplate reader. The average control values were 135 pg/mL and the results were expressed as fold change from control.

### Evaluation of barrier integrity by permeability measurement

The capacity of UDCA and GUDCA to modulate permeability was evaluated in cells treated with UCB for 48 h, the time-point resulting in the maximum disruption of the integrity state of HBMEC monolayer by UCB (Palmela et al., 2012).

In our previous studies, we found that UCB increases the permeability to sodium fluorescein (Palmela et al., 2012), a low molecular weight tracer (376 Da), but not to albumin-bound Evans blue, a high molecular weight tracer (68 kDa). So, in this study HBMEC paracellular permeability assay was conducted with sodium fluorescein as previously described (Veszelka et al., 2007; Cardoso et al., 2012; Palmela et al., 2012). Briefly, cell culture inserts were transferred to 12-well plates containing Ringer–Hepes solution (118 mM NaCl, 4.8 mM KCl, 2.5 mM CaCl_2_, 1.2 mM MgSO_4_, 5.5 mM d-glucose, 20 mM Hepes, pH 7.4) in the basal compartments. The sodium fluorescein solution (10 mg/mL sodium fluorescein in Ringer–Hepes) was added to the upper chambers. The inserts were transferred to new wells at 20, 40, and 60 min. Lower chamber solutions were collected to determine sodium fluorescein levels (Hitachi F-2000 fluorescence spectrophotometer, excitation: 440 nm and emission: 525 nm). Flux across cell-free inserts was also measured. The endothelial permeability coefficient was calculated as previously described (Deli et al., 2005) and the average control permeability coefficient was 1.4 × 10^−5^ cm/s.

### Assessment of UDCA and GUDCA passage across the HBMEC monolayer

To establish whether UDCA and GUDCA are able to cross the BBB endothelium, a two-chamber culture system was used. Bile acids were added to the upper chambers and media from the lower chambers were collected after 4 and 48 h of incubation. The bile acid passage across the HBMEC monolayer was evaluated by measuring the concentrations of UDCA and GUDCA by an enzymatic-fluorimetric assay (Brites et al., 1998). Results were shown as average concentration (μM) ± SEM.

### Statistical analysis

Results are expressed as means ± SEM values from, at least, three separate experiments. Differences between groups were determined by one-way ANOVA with Bonferroni post-test, using Prism 5.0 (GraphPad Software, San Diego, CA). Statistical significance was considered when *P*-values were lower than 0.05.

## Results

### UDCA and GUDCA protect HBMEC from UCB-induced apoptosis, but only GUDCA is effective in reducing caspase-3 activation

**Figure 1:**
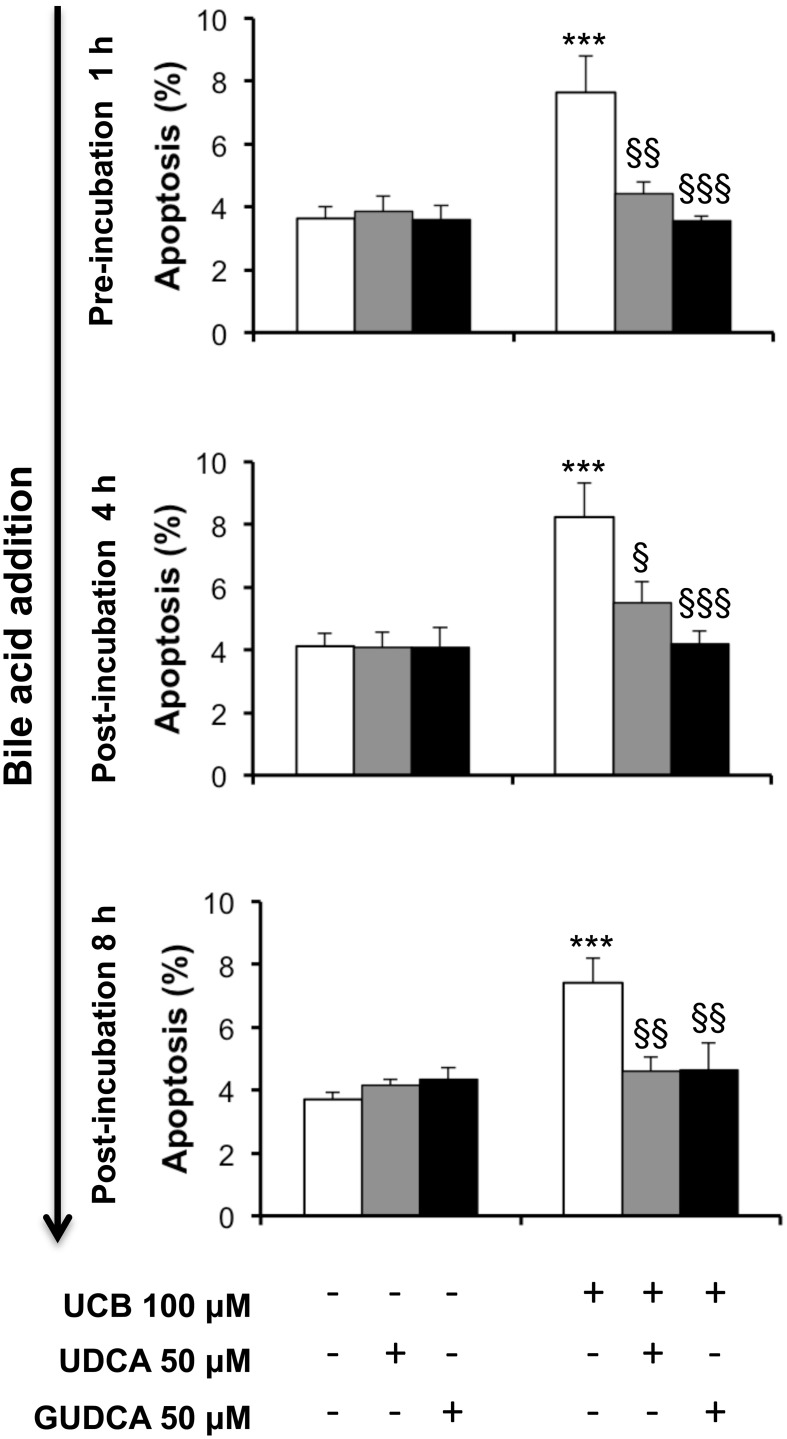
**Ursodeoxycholic acid (UDCA) and glycoursodeoxycholic acid (GUDCA) protect human brain microvascular endothelial cells (HBMEC) from unconjugated bilirubin (UCB)-induced apoptosis**. A cell line of HBMEC was incubated without (control) or with 100 μM UCB, in the presence of 100 μM human serum albumin, for 48 h. The bile acids (50 μM) were added prior to (1 h) or after (4 or 8 h) UCB addition. Quantification of apoptosis is shown as percentage of apoptotic nuclei per total number of cells and results are expressed as mean ± SEM from at least three independent experiments. ^***^P < 0.001 vs. control; ^§^*P* < 0.05, ^§§^*P* < 0.01, and ^§§§^*P* < 0.001 vs. UCB alone.

UCB-induced apoptosis in the HBMEC line includes the presence of apoptotic features that increased with the time of exposure and reached maximal levels at 48 h (Palmela et al., 2011). Thus, this time was chosen to evaluate the ability of UDCA and GUDCA to protect HBMEC from UCB-induced apoptosis. The bile acids were added at three different time points, evaluating their potential when added before and after the injury. Addition of UDCA and GUDCA reduced UCB injury, regardless of the time of addition (Figure 1). This protective effect was maximal in the treatments with GUDCA, especially when added at 1 h prior to UCB addition (54% reduction from UCB values, *P* < 0.001, *vs*. 42% for UDCA at the same time point, *P* < 0.01). Importantly, bile acids partially reverted UCB injury with a nearly 30% protection rate reduction compared to UCB damage (Figure 1), when added 8 h after UCB addition.

**Figure 2:**
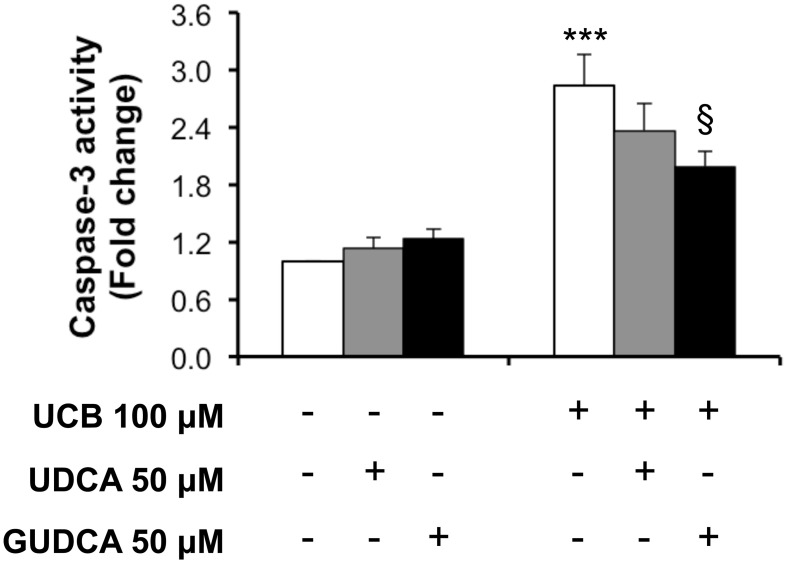
**Glycoursodeoxycholic acid (GUDCA), but not ursodeoxycholic acid (UDCA), has a protective effect on unconjugated bilirubin (UCB)-induced caspase-3 activation in human brain microvascular endothelial cells (HBMEC)**. A cell line of HBMEC was incubated without (control) or with 100 μM UCB, in the presence of 100 μM human serum albumin, for 4 h. The bile acids (50 μM) were added 1 h prior to UCB incubation. Quantification of caspase-3 activity is shown as fold change from control values and results are expressed as mean ± SEM from at least three independent experiments. ^***^P < 0.001 vs. control; ^§^*P* < 0.05 vs. UCB alone.

We next assessed the preventive bile acid effect on caspase-3 activity at 4 h, the time point where the maximal effect of UCB on endothelial cells was observed (Palmela et al., 2011). As seen in Figure 2, only GUDCA was able to significantly protect from UCB-induced activation of caspase-3 (*P* < 0.05).

### Ultrastructural changes induced by UCB in HBMEC are abrogated by UDCA and GUDCA

**Figure 3:**
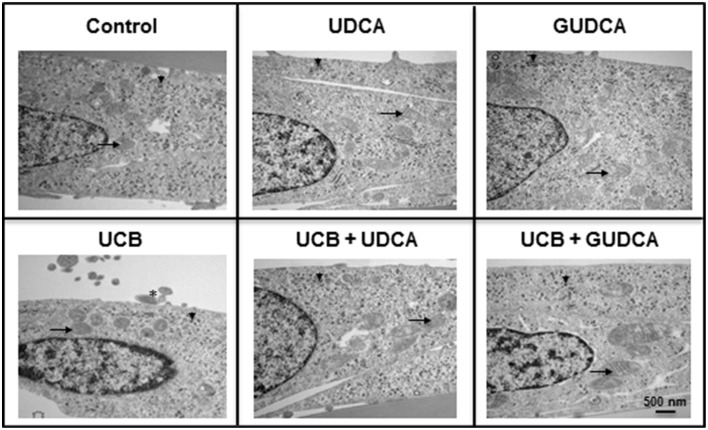
**Ursodeoxycholic acid (UDCA) and glycoursodeoxycholic acid (GUDCA) have protective effects on unconjugated bilirubin (UCB)-induced ultrastructural changes in human brain microvascular endothelial cells (HBMEC)**. A cell line of HBMEC was incubated without (control) or with 100 μM UCB, in the presence of 100 μM human serum albumin, for 48 h. The bile acids (50 μM) were added 1 h prior to the addition of UCB. Representative ultrastructure observations by transmission electron microscopy are shown. In UCB-treated cells note the decrease in mitochondrial cristae (arrows) and ribosomes (arrowhead) and the appearance of detaching cellular fragments (asterisk), which are abrogated by UDCA and GUDCA.

Based on the UCB-induced effects on apoptosis after 48 h incubation and the rescue ability of both UDCA and GUDCA to partially restore cell functionality, we decided to further assess whether changes at HBMEC ultrastructural level were produced by UCB and prevented by bile acid treatment. The transmission electron microscopy analysis revealed a marked reduction in the amount of ribosomes in UCB-treated cells, with an evident recovery in the presence of both bile acids (Figure 3). The same occurred relatively to cellular fragments detaching from the cultured HBMEC and mitochondrial cristae disruption observed, showing the damaging effects of UCB, once again markedly reduced in the presence of each of the bile acids.

### UCB-induced increase of interleukin-6 mRNA and cytokine expression in HBMEC is more effectively reduced by UDCA than by GUDCA

**Figure 4:**
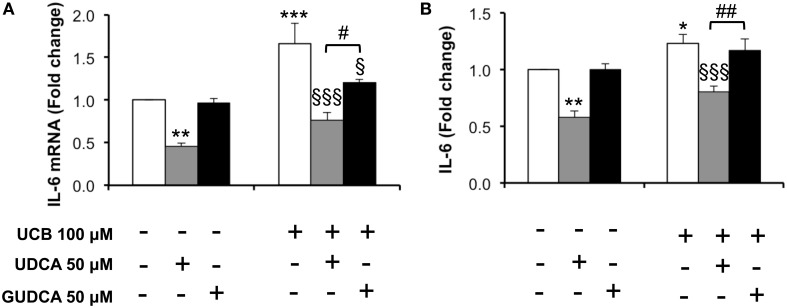
**Decrease in unconjugated bilirubin (UCB)-induced interlkeukin (IL)-6 mRNA expression and cytokine release in human brain microvascular endothelila cells (HBMEC) is higher with ursodeoxycholic acid (UDCA) than with glycoursodeoxycholic acid (GUDCA)**. A cell line of HBMEC was incubated without (control) or with 100 μM UCB, in the presence of 100 μM human serum albumin, for 1 or 4 h. The bile acids (50 μM) were added 1 h prior to the addition of UCB. Changes in IL-6 mRNA after 1 h **(A)** and protein release after 4 h of incubation **(B)** are shown. Quantification is shown as fold change from control values and results are expressed as mean ± SEM from at least three independent experiments. ^*^P < 0.05, ^**^P < 0.01, and ^***^P < 0.001 vs. control; ^§^*P* < 0.05 and ^§§§^*P* < 0.001 vs. UCB alone; ^#^*P* < 0.05 and ^##^*P* < 0.01 UDCA vs. GUDCA.

One of the important effects of UCB on HBMEC previously observed by us was the upregulation of interleukin-6 mRNA levels and protein secretion (Palmela et al., 2011). This previous work indicated that UCB induced the maximum cytokine secretion at 4 h, while the highest mRNA expression was at 1 h following UCB exposure. These time points were then selected and the bile acids were added 1 h prior to UCB incubation. As seen in Figure 4, both bile acids abrogated interleukin-6 mRNA upregulation (Figure 4A), with reductions from UCB values of 27% for GUDCA (*P* < 0.05) and 46% for UDCA (*P* < 0.001). On the other hand, only UDCA showed preventive effects on UCB-induced release of interleukin-6 (Figure 4B), decreasing UCB-induced cytokine secretion by 35% (*P* < 0.001).

### UDCA and GUDCA prevent and rescue disruption of HBMEC integrity by UCB

**Figure 5:**
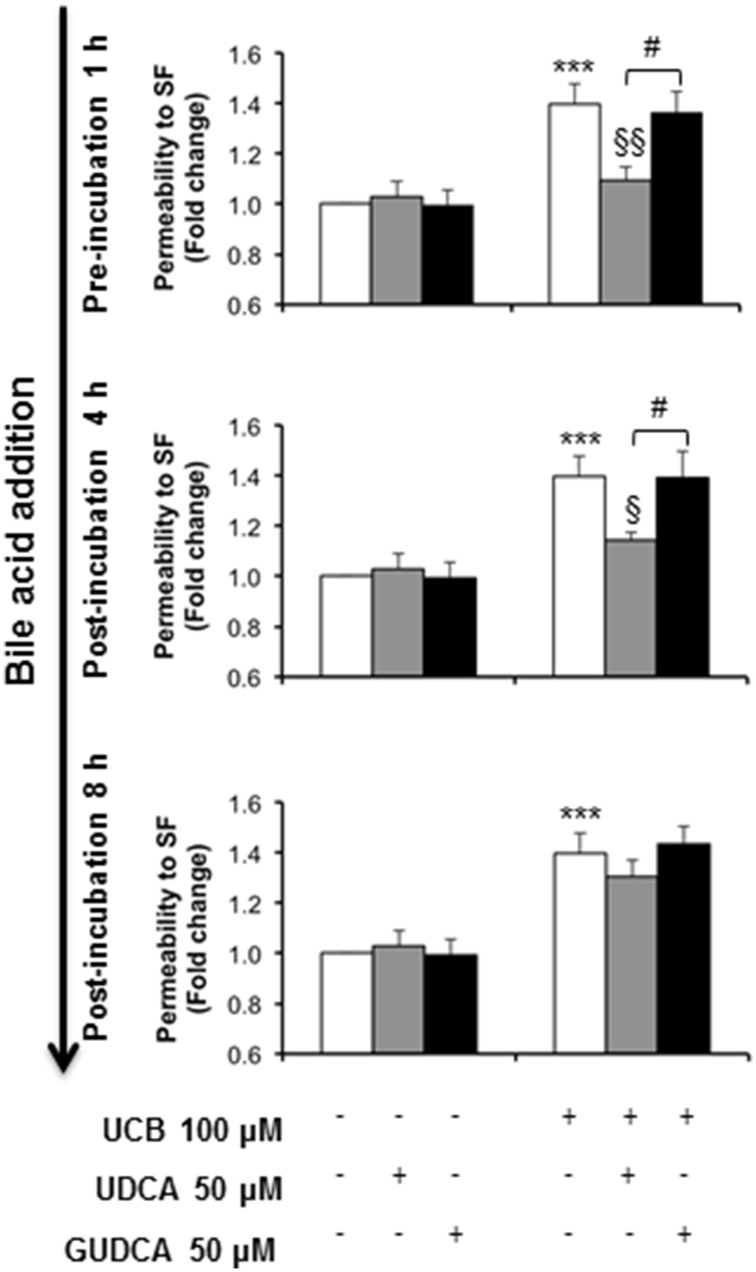
**Ursodeoxycholic acid (UDCA), but not glycoursodeoxycholic acid (GUDCA), abrogates paracellular permeability impairment to sodium fluorescein (SF) in human brain microvascular endothelial cells (HBMEC)**. A cell line of HBMEC was incubated without (control) or with 100 μM UCB, in the presence of 100 μM human serum albumin, for 48 h. The bile acids (50 μM) were added prior to (1 h) or after (4 or 8 h) UCB addition. Quantification of permeability to SF is expressed as fold change from control values and results are shown as mean ± SEM from at least three independent experiments. ^***^P < 0.001 vs. control; ^§^*P* < 0.05 and ^§§^*P* < 0.01 vs. UCB alone; ^#^*P* < 0.05 UDCA vs. GUDCA.

The ability of the tested bile acids to counteract UCB-induced upregulation of interleukin-6, led us to hypothesize that UDCA and GUDCA would protect against the consequent endothelial hyperpermeability. Thus, we measured the paracellular permeability to the low molecular weight compound, sodium fluorescein. This is a widely used indicator of the barrier properties, with several studies showing increased values in conditions associated with hyperpermeability (Hülper et al., 2013; Labus et al., 2014). In our previous study we showed that this parameter is significantly enhanced upon prolonged UCB exposure (Palmela et al., 2012), as also observed in the present study (Figure 5). Here, we also observed that UDCA and GUDCA alone do not affect the HBMEC integrity, since we did not observe any changes in permeability values. However, analysis of the bile acids effect on the permeability to sodium fluorescein revealed that only UDCA prevented UCB injury, and if added before (22% reduction from UCB values, *P* < 0.01) or at 4 h (18% protection from UCB values, *P* < 0.05). In fact, while UCB induced an increased passage of sodium fluorescein molecules from 1.42 × 10^−5^ cm/s in controls to 2.48 × 10^−5^ cm/s in UCB-treated samples, incubation with UDCA reduced such value to 1.95 × 10^−5^ cm/s or to 1.99 × 10^−5^cm/s in cells pre-treated or treated 4 h after UCB addition. In contrast, the values obtained for GUDCA were 2.19 × 10^−5^ and 2.24 × 10^−5^ cm/s (pre- and 4 h after UCB addition treatments, respectively), thus not different from UCB values.

### UDCA and GUDCA cross the HBMEC monolayer in a time-dependent manner

The addition of 50 μM of each of the studied bile acids to the upper (“blood”) compartment of an insert culture system was performed to evaluate if they were able to cross the HBMEC monolayer and thus hypothetically achieve the brain parenchyma. After a short period of incubation (4 h) the bile acids were barely detectable in the lower chamber of the culture plate. However, when longer periods of treatment were applied (48 h) a significant increase in the bile acid passage through the monolayer was obtained (18.8 ± 4.8 and 16.2 ± 3.9 μM, for UDCA and GUDCA, respectively).

## Discussion

In this study we investigated the ability of the bile acid UDCA and its glycine conjugate GUDCA to abrogate the injury caused by UCB in a simplified *in vitro* model of the human BBB, formed by confluent monolayers of HBMEC. The beneficial role of UDCA and its conjugates on liver-associated pathologies has been extensively addressed in the past (Lazaridis et al., 2001; Paumgartner and Beuers, 2004; Perez and Briz, 2009). Interestingly, it was also demonstrated that these bile acids act as pleiotropic agents and can be used as therapeutic molecules in other non-hepatic pathological conditions, including tumors, hemorrhagic stroke, and neurodegenerative disorders like amyotrophic lateral sclerosis (Min et al., 2012), Huntington's (Keene et al., 2002), Alzheimer's (Solá et al., 2003), and Parkinson's diseases (Duan et al., 2002). We have previously shown that UDCA and/or GUDCA have protective properties in reducing the UCB-mediated induction of cell death in both neurons and astrocytes (Silva et al., 2001b; Fernandes et al., 2007b; Vaz et al., 2010). Moreover, we observed the beneficial effects of these bile acids in reducing the increased secretion of pro-inflammatory cytokines by astrocytes and microglia treated with UCB (Fernandes et al., 2007b; Silva et al., 2012) and the oxidative injury in neurons exposed to UCB (Brito et al., 2008), as reviewed by Brites (2012). Importantly, numerous studies have demonstrated that these specific therapeutic properties of UDCA and its conjugates do not apply to the more hydrophobic bile acids. In fact, deoxycholic acid (hydrophobic bile acid) was shown to increase lipid polarity and fluidity, while UDCA and TUDCA (hydrophilic bile acids) are able to reverse such effects (Solá et al., 2002). Additionally, UDCA demonstrated to protect neurons from UCB toxicity, while other hydrophobic bile acids like cholic and chenodeoxycholic acids even aggravated UCB injury (Silva et al., 2001b). The same was observed with endothelial cells where deoxycholic and taurochenodeoxycholic acids caused the cell lysis by acting as detergents (Greenwood et al., 1991) while TUDCA revealed beneficial effects in preventing cell damage by other injurious conditions (Viana et al., 2009; Vladykovskaya et al., 2012). Thus, we aimed to understand if the unconjugated species UDCA and the most predominant conjugate GUDCA derivative, with LogP values of 3.0 and 2.02, respectively (Roda et al., 1990), in humans had protective properties in our simplified *in vitro* model of the BBB in conditions mimicking severe hyperbilirubinemia.

Our previous studies have shown that the BMEC response to UCB is extremely elaborate and far more complex than formerly thought. Our *in vitro* findings include the elevation of endothelial cell death, the upregulation of caveolae and caveolin-1 levels, the increased production of interleukin-6, and the release of matrix metalloproteinases (Palmela et al., 2011, 2012; Cardoso et al., 2012). Consequently, these events led to junction weakness, as well as disruption of endothelial barrier integrity. To such disruption may also contribute the impairment of the cytoskeleton organization induced by UCB (Silva et al., 2001a, 2006), which is known to compromise the intercellular junctions assembly and lead to an increased paracellular permeability (Cardoso et al., 2010). Thus, in addition to diffusion of free bilirubin across the brain microvascular endothelium, the paracellular hyperpermeability of the microvasculature may further favor the freely movement of free bilirubin, as well as of UCB-bound to albumin, via the paracellular space. Also interesting is the increased caveolin-1 expression and the enhanced number of caveolae (Palmela et al., 2012) since caveolae are known to transport albumin, which raises the possibility of augmented entrance of albumin-bound UCB by the transcellular pathway. Validating these *in vitro* findings, signs of BBB compromise were also observed in some brain regions of human cases of neonatal kernicterus, including albumin presence in the brain parenchyma, increased vascularization and microvascular hyperpermeability (Brito et al., 2012, 2013), reduced pericyte vascular coverage and loss of basement membrane components (Palmela et al., submitted). These findings suggest that the BBB plays an important role in the progression of brain damage by severe hyperbilirubinemia and, thus, that this interface should be considered when studying this pathology.

Here, we intended to evaluate the effects of UDCA and GUDCA at the time-point corresponding to the most significant UCB-induced injury previously observed. Furthermore, as UCB effects on HBMEC were also concentration-dependent, we focused on the concentration with the most disruptive potential regarding the integrity of HBMEC (100 μM UCB in the presence of 100 μM human serum albumin), which corresponds to the bilirubin:albumin ratio that induces acute bilirubin encephalopathy and kernicterus (Brito et al., 2012; Iskander et al., 2014) and to free bilirubin values found in jaundiced neonates (Brito, 2001; Ahlfors et al., 2009). To assess the therapeutic window of opportunity of GUDCA and UDCA we tested bile acid efficacy when used before (prevention), or at 4 and 8 h after UCB addition (recovery). To further understand if such bile acids can be promising candidates to rescue neural function in brain diseases we assessed their capacity to cross the HBMEC monolayer, here used as an *in vitro* model of BBB.

Bile acids revealed a high capacity in protecting HBMEC from UCB-induced apoptosis. Interestingly, GUDCA was the most effective against apoptotic features and caspase-3 activation, and was able to restore basal levels, even when the injury was already initiated. Although the anti-apoptotic role of UDCA has been documented in hepatic and non-hepatic cells (Amaral et al., 2009a), the findings here reported are the first in a simplified *in vitro* model of the human BBB. Interestingly, apoptotic cell death results in increased permeability (Erdbruegger et al., 2006), rendering conceivable that the mechanisms underlying HBMEC protection may involve the anti-apoptotic properties of the bile acids. Regardless of the mechanism(s) involved in the protection, the present study opens new avenues for treatment of neurodegenerative diseases that have increasingly been associated with endothelial demise and BBB disruption (Zlokovic, 2008).

When analysing the possible ultrastructural changes produced by UCB and their recovery by the bile acids, we noticed that the loss of ribosomes in UCB-treated samples was reverted by both UDCA and GUDCA. Alterations in ribosomes were associated with apoptosis (Nishida et al., 2002) and autophagy (Cebollero et al., 2012), phenomena already observed in HBMEC treated with UCB (Palmela et al., 2011, 2012). Indeed, Hansen et al. (2001) found elevated levels of bilirubin in ribosomes following exposure of rats to hyperbilirubinemia and hyperosmolality, recognized as a risk factor of kernicterus by increasing BBB permeability (Wennberg, 2000). Therefore, it is tentative to speculate that UCB may induce HBMEC ribophagy, a recent term do designate autophagic turnover of ribosomes (Cebollero et al., 2012), and that both bile acids are able to prevent and recover such event from occurring.

The transmission electron microscopy analysis provided further information about the toxicity of UCB to other cell organelles, particularly the mitochondria. Our observations highlighted a loss of mitochondrial cristae after UCB exposure, which appeared to be restored in HBMEC pre-incubated with each of the bile acids. In a recent study, damaged mitochondria in the presence of UCB were identified in rat BMEC (Cardoso et al., 2012). Mitochondria has been considered one of the first targets of UCB injury to the cells (Mustafa and King, 1970), showing accumulation of glycogen, a sign of impaired energetic function (Batty and Millhouse, 1976). Other studies, including several from our own group, have shown loss of mitochondrial membrane potential, release of cytochrome *c*, and impaired cytochrome *c* oxidase activity (Rodrigues et al., 2000, 2002a; Malik et al., 2010; Barateiro et al., 2012). To note that the protective ability of the bile acids, especially UDCA, in preventing mitochondria dysfunction by UCB was shown in some of those studies (Rodrigues et al., 2000, 2002a). Moreover, the mitochondria enlargement that seems to occur in cells treated with GUDCA and UCB may represent a mechanism to protect cells from apoptotic stimuli (Chiche et al., 2010). Detachment of cellular fragments in UCB-treated samples is in line with the release of small vesicles from HBMEC already noticed by scanning electron microscopy (Palmela et al., 2012). All these features corroborate the previously demonstrated interaction of UCB with membranes (Brites and Brito, 2012) and the stabilizing effect of the bile acids at this level (Rodrigues et al., 2002b; Solá et al., 2002).

Amongst the cytokines produced by HBMEC upon UCB interaction is interleukin-6 (Palmela et al., 2011), a cytokine that has been reported to induce the disruption of the BBB (Maruo et al., 1992; De Vries et al., 1996). Only UDCA was able to reduce both interleukin-6 mRNA expression and protein release. Compromised BBB by vascular leak and tight junction disassembly may lead to deregulated flux of molecules and loss of brain homeostasis. Thus, the restorative effects of the bile acids in interleukin-6 levels may contribute to sustain HBMEC integrity in the presence of UCB. While GUDCA revealed strong anti-apoptotic effects, UDCA was more effective in preventing the increased interleukin-6 secretion by HBMEC treated with UCB. Interestingly, UDCA was also more able than GUDCA in rescuing HBMEC from the increased permeability induced by UCB, an effect that was observed even if the bile acid was added 4 h after UCB-addition. Such dissimilarities among the bile acids species may derive from the different changes that UDCA and the conjugated species were shown to produce in the composition of membrane lipid content (Bellentani et al., 1996).

Crossing the BBB is one of the main milestones for therapeutic molecules that are meant to act in the brain. Although detection of UDCA and its taurine conjugate was found in the brain parenchyma after injection in rodents (Kaemmerer et al., 2001; Rodrigues et al., 2003; Parry et al., 2010) and in the cerebrospinal fluid after oral administration in patients with amyotrophic lateral sclerosis (Parry et al., 2010), no studies were till now performed using HBMEC. Here, we show that both UDCA and GUDCA cross the HBMEC monolayer, *in vitro*. However, the mechanism underlying the passage of the bile acids across the BBB has never been reported. Among the factors that may influence BBB permeation are the bile acids' physico-chemical characteristics, namely their octanol/water partition coefficient (Roda et al., 1990), and the presence of several transporters at the BBB (Abbott et al., 2010) that may modulate their passage across the endothelium. Therefore, additional studies are necessary to establish the mechanism involved in these bile acids passage across the BBB. The suggested potential therapeutic role for UDCA and GUDCA also needs further evaluation in animal models of severe jaundice, such as the Gunn rat (Gunn, 1938) or the glucuronosyl-transferase knock-out mice (Nguyen et al., 2008).

Collectively, our *in vitro* data show that the disruption of endothelial cell function and BBB dynamic properties by exposure to UCB in conditions mimicking a severe neonatal jaundice can be prevented and partially restored by UDCA and GUDCA. The results also show a higher efficacy when the bile acids are administered before injury, reinforcing their preventive effects. They further show that both molecules slowly permeate across the BBB endothelium, which points to their potential to reach the brain and elicit therapeutic properties in target cells.

## Author contributions

IP, performed most of the experimental work, analyzed data and drafted the manuscript; LC, RFMS, HS, and KSK contributed to the acquisition, analysis and interpretation of the data, as well as to the review of the manuscript. DB contributed to the analysis and interpretation of data, and critically revised the manuscript. MAB designed the work, contributed to the acquisition, analysis and interpretation of data, and critically revised the manuscript. All the authors approved the version to be published and provided agreement to be accountable for all aspects of the work.

### Conflict of interest statement

The authors declare that the research was conducted in the absence of any commercial or financial relationships that could be construed as a potential conflict of interest.

## Abbreviations

BBB: Blood-brain barrier
GUDCA: Glycoursodeoxycholic acid
HBMEC: Human brain microvascular endothelial cell
IL: Interleukin
SF: Sodium fluorescein
TUDCA: Tauroursodeoxycholic acid
UCB: Unconjugated bilirubin
UDCA: Ursodeoxycholic acid.
